# Mental health measures of anxiety and depression in patients with retinal detachment

**DOI:** 10.1186/1745-0179-3-10

**Published:** 2007-07-19

**Authors:** Maneli Mozaffarieh, Stefan Sacu, Thomas Benesch, Andreas Wedrich

**Affiliations:** 1 Department of Ophthalmology, Medical University of Vienna, Austria; 2 Institute of Medical Statistics, Medical University of Vienna, Austria; 3 Department of Ophthalmology, Medical University of Graz, Austria

## Abstract

In this study, the researchers examined anxious and depressive symptoms of patients with rhegmatogenous retinal detachment (RRD) prior to and up to year after retinal detachment surgery. One hundred and thirteen (113) patients with RRD took part in this prospective longitudinal study. Anxiety and depression were evaluated using the Hospital Anxiety and Depression Scale (HADS). Visual acuity (VA) results and HADS scores of all participants were recorded prior to and 3, 6 and 12 months after retinal detachment surgery. Pearson correlation analysis showed a significant association between the patients' VA and HADS psychological scores both prior to and three months after surgery, regardless of the type of surgery performed. Psychological distress is a significant problem associated with retinal detachments that requires more attention.

## Background

Retinal detachment (RD) is a condition which affects approximately one in 300 patients in the course of a lifetime and more often requires urgent surgical repair [1]. Despite the knowledge on visual outcomes of the patients with retinal detachment, and the multiple reports on outcomes after various surgical interventions [1-6], little is known on the effect of retinal detachment on the patients' psychological status.

It is recognized that psychological symptoms accompany visual loss [7,8]. However, most research in this domain has focused on blind individuals and only little information is available concerning psychological distress among visually impaired (but not blind individuals). In a study done on pre-school children treatment for unilateral visual impairment (amblyopia) was shown to be associated with some degree of psychological distress [9]. Reports suggest that a large number of people with advanced age-related macular degeneration have depressive disorders leading to serious consequences for their quality of life [10-12]. The impact of ocular surgery on patient's psychological reactions is also limited to certain surgical interventions. Patients for cataract surgery, for example, suffer from psychological disorders of anxiety and depression after surgery, in particular if their vision has not improved significantly [13]. A study on patients with uveal melanoma reported a prevalence of mental disorders of anxiety and depression after eye surgery [14]. Unfortunately, to date, there have been no studies investigating whether RD has any effect on the patients' psychological status. The lack of familiarity with the psychological consequences of acute visual loss secondary to RD prevents most physicians from supplying these patients with optimal additional treatment and support. As a result, the psychological symptoms which accompany RD are overlooked in many patients.

The purpose of this article is to examine the psychological reactions of patients with RD prior to and after the successful surgical intervention. To our knowledge this is the first study seeking to find the impact of RD on the patients' psychological status.

## Methods

### Participants

One hundred and thirteen patients (113 eyes) with RD were included in this prospective longitudinal clinical study. The study was performed at the Department of Ophthalmology at the Vienna General Hospital (Medical University of Vienna) between 2000 and 2003. The inclusion criteria included the identification of rhegmatogenous detachment by a vitreoretinal specialist. Patients with glaucoma, uveitis or any other coexisting visually limiting condition (e.g. excudative age-related macular degeneration) or RRD with a proliferative vitreoretinopathy stage 3 or 4 were excluded from the study. Additionally, patients with potentially confounding events (e.g death of family member) that may have influenced overall psychological stress were excluded from the study (1 patient). The operation was considered anatomically successful if the retina was still re-attached at least six months after the operation. Patients with re-detachment during the study period for second surgical repair were excluded from the study. Written informed consent was obtained from all patients after their involvement in the study had been explained in detail.

### Surgical Procedure

All surgery was performed by an exprienced vitreoretinal surgeon. The surgeon (AW) involved in the study decided the surgical approach in each case according to his experience. Patients underwent either a vitrectomy with fluid-gas exchange or scleral buckling surgery under general anaesthesia.

### Visual Acuity

Visual acuity was recorded as the best corrected visual acuity in the study eye and the fellow eye using a Snellen chart prior to and three, six and twelve months after surgery. The Snellen fractions were converted to a log scale (Log MAR) as previously described [15].

### The Hospital Anxiety and Depression Scale (HADS)

The hospital anxiety and depression scale was developed to measure anxiety and depressive symptoms among patients with somatic diseases [16]. The reliability and validity of the HADS have been tested in a number of international studies [17]. The HADS contains 14 items and consists of two subscales: 7 items for anxiety and 7 for depression. Each item is rated on a four-point scale (scored 0–3), giving maximum scores of 21 for anxiety and depression. This questionnaire was administered to the patients prior to, and 3, 6 and 12 months after surgery.

### Statistical Analysis

Using SPSS 12.0 for Windows, the descriptive and inferential data analyses were performed. Mean visual acuity and HADS psychological scores were evaluated using a paired t-test for change from baseline. Analysis of variance (ANOVA) was used for between types of surgical treatments evaluation (vitrectomy/buckle surgery) and for subgroup analysis based on gender of patients (female/male). For nonparametric data, the Mann-Whitney U test was applied. Univariate associations between mean visual acuity and HADS psychological scores were evaluated using the Pearson correlation test. The probabilities were all corrected for multiple testing, applying the Bonferroni-Holm step-down test (α = 0.05).

## Results

### Descriptive data of the study population

A total of 113 patients with RD were eligible according to our inclusion criteria. Of these ten patients were not included (5 did not complete questionnaires, 4 did not attend their follow-up's, and 1 lived abroad). Follow-up data were available for the remaining 103 patients who were studied prospectively. The mean age of the patients 56.5 years (SD, ± 11.6 years; range: 27–81). Fifty-two (50%) of the patients were male and 51 (50%) were female. 65 of the patients (63%) had vitrectomy with fluid-gas exchange and 38 (37%) had buckle surgery.

### Changes from baseline for all parameters

The mean best corrected visual acuity (Log MAR) of the patients prior to and 3, 6 and 12 months after surgery, was 1.3 ± 0.2, 0.62 ± 0.06, 0.43 ± 0.04 and 0.26 ± 0.06, respectively (p < 0.05 to baseline, table 1). The mean best corrected visual acuity of the patients fellow-eye prior to and 3, 6 and 12 months after surgery, was 0.21 ± 0.09, 0.19 ± 0.07, 0.20 ± 0.06, and 0.19 ± 0.06, respectively (p > 0.05 to baseline).

**Table 1:** Visual acuity (Log MAR) and hospital anxiety and depression scores (HADS)

	Baseline	3 months after surgery		6 months after surgery		12 months after surgery	
	
	Mean ± SD	Mean ± SD	P*	Mean ± SD	P*	Mean ± SD	P*
**Study eye Log MAR**	1.3 ± 0.2	0.62 ± 0.06	< 0.05	0.43 ± 0.04	< 0.01	0.26 ± 0.06	< 0.01
**Fellow-eye Log MAR**	0.21 ± 0.09	0.19 ± 0.07	> 0.2	0.2 ± 0.06	> 0.2	0.19 ± 0.06	> 0.2
**Anxiety**	18 ± 3	17 ± 3	< 0.01	16 ± 3	< 0.01	16 ± 3	< 0.01
**Depression**	16 ±	13 ± 2	< 0.01	8 ± 3	> 0.1	5 ± 2	> 0.1

* for change from baseline

Means and standard deviations (SD) of visual acuity and Hospital Anxiety and Depression Scores (HADS) at the 4 points of assessment.

The cut-off values for anxious and depressive moods are depicted in table 2. Patients were significantly more psychologically affected prior to than after detachment surgery (HADS anxiety and depression, p < 0.01). More than half of the patients (65%) experienced 'probable' levels of anxiety, prior to surgery. This value decreased to 59.2%, 12 months after RRD surgery. Percent of patients psychologically affected and mean HADS scores are presented in table 2.

**Table 2:** Hospital anxiety and depression scores (HADS) of patients prior to and after retinal detachment surgery

	Prior to surgical treatment N (%)	3 months after treatment N (%)	6 months after treatment N (%)	12 months after treatment N (%)
**Anxiety**				
**0–7**	6 (5.8)	9 (8.7)	11 (10.6)	13 (12.6)
**8–10**	67 (65.0)	60 (58.2)	70 (67.9)	61 (59.2)
**> 10**	30 (29.1)	29 (28.1)	27 (26.2)	29 (28.1)
**Depression**				
**0–7**	31 (30.1)	57 (55.3)	72 (69.9)	82 (79.6)
**8–10**	52 (50.5)	31 (30.1)	22 (21.3)	16 (15.5)
**> 10**	20 (19.4)	15 (14.5)	9 (8.7)	5 (4.8)

Number (%) of patients scoring in categories 'No case' (< 7), 'Possible clinical case' (8–10) and 'Clinical case' (> 10) on the Hospital Anxiety and Depression Subscales. A score of 0 to 7 for either subscale of depression or anxiety is regarded as being in the normal range, a score of 8 to 10 indicates probable presence of the mood disorder, and a score larger of 10 or larger is suggestive of the clinical presence of the mood disorder.

### Subgroup analysis

No statistically significant difference was found between the type of surgery performed (vitrectomy or buckle) and HADS psychological scores at any follow-up time (p > 0.05). The patients' gender had no statistically significant effect on HADS psychological scores (p = 0.31).

### Association between the visual acuity and the psychological reactions

#### Study eye

There were strong correlations between the visual acuity and HADS psychological scores for depression and anxiety 3 months after surgery (r_3A _= 0.68, r_3D _= 0.74, p < 0.01). Moderate correlations were found between the visual acuity and HADS psychological scores for anxiety both six and twelve after surgery (r_6A _= 0.45, r_12A _= 0.39, p < 0.01). No significant association was found between the visual acuity and HADS depression scores of the patients' either at 6 or 12 months (r_6D _= 0.35, r_12D _= 0.26 p > 0.05).

#### Fellow-eye

Interestingly, there was a significant association between the patients' fellow-eye visual acuity and HADS anxiety scores both 6 and 12 months after surgery (r_6 _= 0.86, r_12 _= 0.74, p < 0.01). No significant association was found between patients' fellow-eye visual acuity and HADS depression scores either 6 or 12 months after surgery (r_6 _= 0.32, r_12 _= 0.21, p > 0.1).

## Discussion

This investigation reports the long-term impact of psychological affects in patients with retinal detachment. The assessment and follow-up of the patients' psychological characteristics were measured by means of the reliable and valid HADS questionnaire. The reliability of this measure has been reported in numerous previous studies making its use internationally applicable [16,17]. We then further sought to observe the relationships between the patients' psychological status and visual acuity prior to and after surgery.

RRD is usually an acute condition for which surgery is generally considered mandatory in order to prevent severe and permanent visual loss. We found that more than half of our patients (65%) experienced probable levels of anxiety, prior to surgery. This value decreased gradually to 59%, 12 months after surgery. Patients were most probably relieved to have had surgery. Still, the prevalence of anxiety, demands our attention as to why and what we could possibly do to alleviate this symptom.

Retinal detachment is accompanied by a certain degree of functional loss which is regained gradually [18]; this may explain the reason for the high HADS anxiety scores. Ophthalmologists may support patients by acting as facilitators providing information about RD and the various surgical interventions, and by suggesting ways to ameliorate anxious symptoms. Patients may, for example, find it alleviating to share their experiences with others who were similarly affected or they may just simply need more reassurance to relieve their concerns.

Anxiety is a multidimensional response to stimuli in the person's environment, or a response to an internal stimulus resulting from a combination of general biological and individual psychological processes. Physicians should be aware that there may be a certain degree of anxiety associated with the fear of similar RRD occurrence in the fellow-eye which may also have substantive negative effects in the patients' overall status. Data from other studies suggest that giving patients' choices about, information, and responsibility for certain aspects of care has important implications for the quality of care and health outcomes [19-21]. Especially patients with RD should be adequately informed on all the consequences of surgery, the status of their fellow-eye and possibly be advised that they are capable of, and may take over daily routine activities.

Unfortunately obstacles such as resource and time constraints or the primary lack of knowledge that indeed patients with RD are psychologically affected, prevent us in our efforts to optimally treat patients. Evidence of interactions between physician and patient suggests that as the pressures to contain costs increase, physicians respond by increasing the volume of their practices with a corresponding decrease in time spent per patient [22]. Busy hospital practices and shorter office visits have been empirically linked with less effective patient behaviour, whereas more time with the physician has been associated with more favourable ratings of the clinical encounter. Our data suggest that more time with patients after surgery may be required to adequately discuss visual and psychological outcomes of vitreoretinal surgery.

A limitation in this study is the fact that this study includes data from patients operated on by one vitreoretinal surgeon at one center. Data from this patient pool may not reflect the outcomes of persons undergoing vitreoretinal surgical procedures by other surgeons at other centers. Another limitation is that although the HADS instrument has been reported to be a very reliable instrument, its reliability was not measured in the current study. HADS is a very useful first step of a screening which must be multiphasic. More data is therefore required to back-up our results firmly. An extensive clinical interview, by professionals, would be an important and necessary second step. In our investigation we evaluated psychological responses of patients with uncomplicated cases of RD; cases requiring additional surgery, which may have been exposed to much more psychological stress, were not included in this study.

Further research should evaluate various psychosocial aspects of RRD and provide patients with suitable therapeutic interventions. If data from future research supports our results, then more attention must be paid to the psychological manifestations of acute visual impairment and to approaches which may improve psychological management to provide optimal care for patients.

## Authors' contributions

MM carried out the study.

SS participated in the design of the study, drafted the study and contributed in the statistical analysis.

TB performed the statistical analysis.

AW conceived of the study, and participated in its design and coordination.
